# Clinical Diagnostic and Prognostic Value of Residual Language Learning Ability in Patients with Disorders of Consciousness

**DOI:** 10.1523/JNEUROSCI.1684-24.2025

**Published:** 2025-04-17

**Authors:** Yan Chen, Xiangyue Xiao, Zhicai Dong, Junhua Ding, Sara Cruz, Ming Zhang, Yuhan Lu, Nai Ding, Charlène Aubinet, Steven Laureys, Haibo Di

**Affiliations:** ^1^International Unresponsive Wakefulness Syndrome and Consciousness Science Institute, Hangzhou Normal University, Hangzhou 311121, China; ^2^Key Laboratory of Ageing and Cancer Biology of Zhejiang Province, School of Basic Medical Sciences, Hangzhou Normal University, Hangzhou 311121, China; ^3^State Key Laboratory of Cognitive Science and Mental Health, Institute of Psychology, Chinese Academy of Sciences, Beijing 100101, China; ^4^The Psychology for Development Research Centre, Lusiada University Porto, Porto 4100-348, Portugal; ^5^Shanghai Yongci Rehabilitation Hospital, Shanghai 201100, China; ^6^Shanghai Key Laboratory of Brain Functional Genomics (Ministry of Education), School of Psychology and Cognitive Science, East China Normal University, Shanghai 200062, China; ^7^Key Laboratory for Biomedical Engineering of Ministry of Education, College of Biomedical Engineering and Instrument Sciences, Zhejiang University, Hangzhou 310027, China; ^8^Coma Science Group, GIGA Consciousness & Centre du Cerveau, University and University Hospital of Liège, Liège 4000, Belgium; ^9^Psychology & Neuroscience of Cognition Research Unit, University of Liège, Liège 4000, Belgium

**Keywords:** diagnosis, disorders of consciousness, electroencephalogram, explicit learning, language, prognosis

## Abstract

Recent research suggests that the detection of preserved cognitive function can assist in the diagnosis and prognosis of patients with disorders of consciousness (DoC). This study investigates EEG signals as indicators of neural activity associated with the processing of transitional probabilities during a learning paradigm in patients with DoC. By examining the sensitivity to transitional probabilities across levels of consciousness, we aim to assess the potential value of this indicator in clinical diagnosis and prognosis. We collected EEG recordings from 51 DoC patients (10 female) and 26 healthy controls (9 female). EEG activity was recorded while participants listened to artificial vocabulary speech sequences before and after the learning phase. Intertrial phase coherence (ITPC) was used to examine differences in neural responses in different learning phases. Results showed that minimally conscious patients showed a significant increase in the word-tracking response after the learning phase, similar to healthy controls. Moreover, their learning-mediated word-rate ITPC difference correlated significantly with their Coma Recovery Scale-Revised score and 6 month outcome. However, these correlations were absent in unresponsive wakefulness syndrome patients. Crucially, differences in vocabulary ITPC before and after the learning phase effectively discriminated between healthy controls and patients, as well as between minimally conscious and unresponsive wakefulness syndrome patients. Combining EEG indicators with clinical performance accurately predicted patients’ prognosis. In conclusion, the language learning paradigm has the potential to contribute to both diagnosis and prognosis in this challenging population, thereby significantly reducing prognostic uncertainty in medical decision-making and benefiting the rehabilitation of DoC patients.

## Significance Statement

This study explores the electroencephalogram sensitivity to changes in transitional probabilities during a learning paradigm, and its relationship to diagnosis and prognosis in patients with disorders of consciousness (DoC). Our results demonstrated that minimally conscious patients exhibited a significant increase in intertrial phase coherence values at word frequencies after the learning phase, similar to healthy controls, suggesting retained language ability. In contrast, patients with unresponsive wakefulness syndrome did not show such improvements. Combining electroencephalogram indicators with clinical assessments in a predictive model could improve the accuracy of diagnosis and prognosis of patients. In sum, this objective measurement of brain responses could reduce the prognostic uncertainty in clinical decision-making and better guide the care and rehabilitation of DoC patients.

## Introduction

Depending on the degree of consciousness impairment, disorders of consciousness (DoC) can be classified into unresponsive wakefulness syndrome (UWS) and minimally conscious state (MCS; Giacino et al., 2014; Gosseries et al., 2014). Patients with UWS may open their eyes spontaneously but show no signs of awareness, while patients with MCS may occasionally show self-awareness or awareness of their environment (Giacino et al., 2014; Gosseries et al., 2014; Tobaldini et al., 2018) and typically have a better prognosis (Hirschberg and Giacino, 2011; Gantner et al., 2012). However, traditional behavioral assessments often fail to capture latent consciousness, particularly in patients with some deficits (e.g., motor dysfunction), leading to high rates of misdiagnosis (Giacino et al., 2018) and subsequent delays in treatment progress (Wannez et al., 2017; Tinti et al., 2024). To address this issue, researchers have turned to objective neuroimaging techniques for complementary insights (Di Perri et al., 2016; Edlow et al., 2021; Hermann et al., 2021). Among these, the electroencephalogram (EEG) stands out for its noninvasive nature, portability, and affordability, making it a valuable tool for clinical assessments to improve diagnosis (Chennu et al., 2017; Engemann et al., 2018).

Emerging evidence suggests that some patients with DoC retain higher-level cognition, despite their impaired behavioral responses. For example, Naci et al. (2014, 2017) and Laforge et al. (2020) reported that some patients showed similar brain activity to healthy controls when exposed to suspenseful audio/visual stimuli, suggesting preserved narrative processing abilities. Furthermore, Aubinet et al. (2022) highlighted the importance of exploring language-related brain activity in patients with DoC to infer their consciousness levels. Several studies have investigated the relationship between linguistic processing and consciousness in this population. Gui et al. (2020) observed that while both patients and healthy controls showed neural responses to syllable frequency in speech stimuli, word- and sentence-rate responses were reduced in MCS patients and absent in UWS patients, indicating a relationship between consciousness levels and linguistic processing depth. Braiman et al. (2018) found patients with UWS exhibited delayed neural responses to natural speech compared with MCS and controls, highlighting differences in language comprehension across DoC subgroups. However, these studies focused on implicit paradigms that reflect automated processing without addressing the capacity for more deliberate language learning. The acquisition of rule-based sequential learning, such as identifying multisyllabic words in a speech stream by tracking transitional probabilities, is considered an evolutionarily conserved skill with important implications for language development (Hauser et al., 2002). An explicit learning paradigm facilitates the acquisition of more abstract semantic knowledge, which generally improves learning outcomes (Takashima et al., 2014, 2017). It involves a conscious, deliberate effort to learn rules or patterns (DeKeyser, 2003; Dörnyei, 2008) and requires awareness of the learning target (Ellis, 1994; DeKeyser, 2003). Research has shown that explicit vocabulary learning can increase the amplitude of vocabulary-related cortical tracking in healthy adults (Chen et al., 2020). Inspired by these findings, we aimed to investigate whether an explicit vocabulary learning environment could induce neural changes in patients with DoC, and how these changes are related to their level of consciousness.

To address these questions, the current study examined neural responses to transitional probabilities at the word level in patients with DoC using an explicit learning paradigm. Patients and healthy controls underwent explicit learning of five artificial words and listened to speech sequences of the artificial words before and after the learning session. By comparing EEG responses before and after the learning sessions, we aimed to evaluate the learning effect in each group as a clinical research objective. In addition, we sought to explore the potential of these EEG-based measures as translational tools for the development of diagnostic and prognostic indicators for DoC patients. We hypothesize that the patients with MCS may exhibit changes in neural responses through an explicit learning paradigm and that their EEG characteristics may reflect this processing, providing insight into patient diagnosis and prognosis.

## Materials and Methods

### Ethics statement

This study was approved by the Ethics Committee of the Hangzhou Normal University. The procedures used in this study adhere to the tenets of the Declaration of Helsinki. Informed consent was obtained from the primary caregivers of the DoC patients.

### Participants

Fifty-one patients with DoC were recruited from the Shanghai Yongci Rehabilitation Hospital and the rehabilitation units of the Hangzhou Wujing Hospital. Inclusion criteria were as follows: (1) diagnosis of MCS or UWS according to the Coma Recovery Scale-Revised (CRS-R; Giacino et al., 2004; Kondziella et al., 2020); (2) age over 18 years; and (3) time since brain injury more than 28 d; (4) presence of the auditory startle reflex. Exclusion criteria were as follows: (1) history of hearing or vision impairment prior to brain injury; (2) history of psychiatric or neurological disease prior to brain injury; (3) obvious cranial bone defects; and (4) sedation within 24 h prior to behavioral or EEG assessment. Five patients were excluded due to poor EEG data quality [four met the exclusion criteria of Gui et al. (2020) and one had a fever during the experiment]. A total of 46 patients were included in the final analysis—25 MCS patients (19 males; mean age 61.24 ± 12.41 years) and 21 UWS patients (18 males; mean age, 52.14 ± 15.64 years). The demographic and clinical characteristics of the two groups of patients are provided in Table 1.

**Table 1.** The demographic and clinical information of 46 patients with DoC

Patient ID	Diagnosis	Age (years)	Gender	Etiology	Time since injury (months)	CRS-R score	Diagnosis at 6 months	Follow-up CRS-R score
MCS01	MCS−	29	M	TBI	10	10	DIED	N/A
MCS02	MCS−	44	M	NTBI	35	11	N/A	N/A
MCS03	MCS−	66	M	TBI	11	12	N/A	N/A
MCS04	MCS−	67	M	TBI	14	10	MCS−	11
MCS05	MCS−	81	M	NTBI	16	11	N/A	N/A
MCS06	MCS−	59	F	NTBI	4	10	MCS−	10
MCS07	MCS+	70	F	TBI	14	19	DIED	N/A
MCS08	MCS−	50	M	NTBI	18	9	MCS−	11
MCS09	MCS+	66	M	TBI	13	11	MCS−	10
MCS10	MCS+	66	F	TBI	15	18	EMCS	20
MCS11	MCS−	53	M	NTBI	8	12	MCS−	13
MCS12	MCS−	67	F	NTBI	12	12	MCS−	10
MCS13	MCS−	77	M	NTBI	20	12	MCS−	7
MCS14	MCS+	72	F	NTBI	10	15	MCS+	15
MCS15	MCS−	51	M	TBI	12	11	MCS−	14
MCS16	MCS−	75	M	TBI	18	14	MCS−	10
MCS17	MCS−	50	M	NTBI	23	11	DIED	N/A
MCS18	MCS−	51	M	NTBI	11	13	MCS−	10
MCS19	MCS−	68	M	NTBI	20	9	UWS	6
MCS20	MCS−	72	M	NTBI	19	12	MCS−	9
MCS21	MCS−	52	M	TBI	9	13	MCS−	13
MCS22	MCS−	66	M	NTBI	16	13	MCS−	12
MCS23	MCS−	64	F	NTBI	18	11	MCS−	9
MCS24	MCS−	71	M	NTBI	22	12	MCS−	7
MCS25	MCS−	44	M	NTBI	10	9	UWS	10
UWS01	UWS	75	M	NTBI	13	7	N/A	N/A
UWS02	UWS	71	M	TBI	10	5	DIED	N/A
UWS03	UWS	37	M	NTBI	11	7	N/A	N/A
UWS04	UWS	34	M	NTBI	12	5	UWS	8
UWS05	UWS	42	M	NTBI	7	7	UWS	8
UWS06	UWS	41	M	NTBI	5	7	UWS	7
UWS07	UWS	33	M	TBI	15	7	UWS	7
UWS08	UWS	55	M	TBI	12	7	MCS−	9
UWS09	UWS	54	M	TBI	3	7	UWS	7
UWS10	UWS	73	M	NTBI	18	2	UWS	5
UWS11	UWS	25	F	NTBI	9	7	UWS	8
UWS12	UWS	52	M	TBI	15	7	N/A	N/A
UWS13	UWS	29	F	NTBI	10	7	UWS	7
UWS14	UWS	71	M	NTBI	32	8	UWS	6
UWS15	UWS	67	M	TBI	26	7	UWS	8
UWS16	UWS	55	M	NTBI	13	7	N/A	N/A
UWS17	UWS	56	M	NTBI	10	6	UWS	7
UWS18	UWS	68	M	TBI	22	7	UWS	6
UWS19	UWS	65	M	NTBI	10	7	UWS	5
UWS20	UWS	51	F	NTBI	16	7	UWS	8
UWS21	UWS	41	M	NTBI	21	7	UWS	6

CRS-R, coma recovery scale-revised; DoC, disorders of consciousness; F, female; M, male; MCS, minimally conscious state without (MCS−) or with (MCS+) evidence of language function; N/A, data not available; NTBI, nontraumatic brain injury; TBI, traumatic brain injury; UWS, unresponsive wakefulness syndrome.

Twenty-six healthy controls (HC) were recruited from the local community. One participant was excluded due to the occurrence of extreme body movements during the experiment. The remaining 25 HC (16 males; mean age, 58.60 ± 11.08 years) were included in the study. They had normal hearing and vision and no history of psychiatric or neurological disease.

All participants were right-handed and native speakers of Mandarin Chinese. There was no significant difference in the age or gender distribution between the HC and the two patient groups (age: one-way ANOVA, *F* = 2.893, *p* = 0.062; gender: chi-square test, *χ*^2^ = 2.881, *p* = 0.237).

### Clinical assessments

All patients underwent five CRS-R assessments by two trained neurologists in the 10 d prior to EEG data collection. The highest score obtained from the five assessments was considered the standard for the diagnosis of MCS or UWS (Wannez et al., 2017). To assess prognostic value, follow-up CRS-R assessments were performed 6 months after EEG data collection. The raters responsible for determining the clinical performance remained blinded to the EEG results throughout the assessment process.

### Stimuli

The stimuli for the learning task consisted of a series of Chinese syllables, forming a 10 min speech sequence of five trisyllabic artificial words. These trisyllabic artificial words (i.e., nonwords or pseudowords) were created by combining five trisyllabic real Mandarin Chinese words that are commonly used in daily life and have practical meanings (i.e., “cháng jǐng lù” for giraffe, “xī hóng shì” for tomato, “tuī xiāo yuán” for salesman, “diàn yùn dǒu” for iron, and “fŭ wò chēng” for push-up). These real words were chosen with the intention of maximizing the difference in the initial phonemes, final phonemes, and tones of the syllables at the same positions in these words. Each syllable appeared in only one real word. By shuffling the syllables at the same position within the real words, five trisyllabic artificial words were created (i.e., “cháng xiāo shì,” “xī wò dǒu,” “tuī jǐng chēng,” “diàn hóng lù,” “fŭ yùn yuán”). These artificial words had no semantic associations or meanings.

The speech stimuli were presented in a male voice using the NeoSpeech synthesizer (http://www.neospeech.com/). Each syllable had a fixed duration of 250 ms. The syllables were concatenated with no pauses or acoustic gaps between them. Each artificial word used in this study consisted of three syllables, resulting in a presentation rate of 1.33 Hz for the word and 4 Hz for the syllable. The constructed speech sequence consisted of 2,400 syllables totally, corresponding to 800 artificial words. The order of presentation of these words was pseudorandomized and the same word was not repeated immediately after its presentation. The transitional probability between neighboring syllables was always 1.0 within each word and 0.25 at word boundaries. To truly assess participants’ learning abilities, we added 7–8 random syllables at the beginning and end of the speech sequence to prevent participants from arbitrarily dividing the sequence into words every three syllables from the beginning. These extra syllables were randomly selected from the syllables of the artificial words. The speech sequence had a total duration of 10 min and 3.75 s. Within this sequence, 10 syllables were modulated by adjusting their formant shift ratio to produce a higher pitch perception and were referred to as “anomalous syllables” for the target detection task. All stimuli were presented using the Psychtoolbox (Brainard, 1997). Although the experimental stimuli contained acoustic/phonological confounds (Extended Data Figs. 1-1, 1-2), the observed experimental effects focused on neural changes before and after the learning phase, which reduced the potential influence of the physical properties of the stimuli.

### Procedures

The EEG experiment was conducted on two consecutive days. Participants were presented with artificial word speech sequences through headphones.

**Figure 1.**
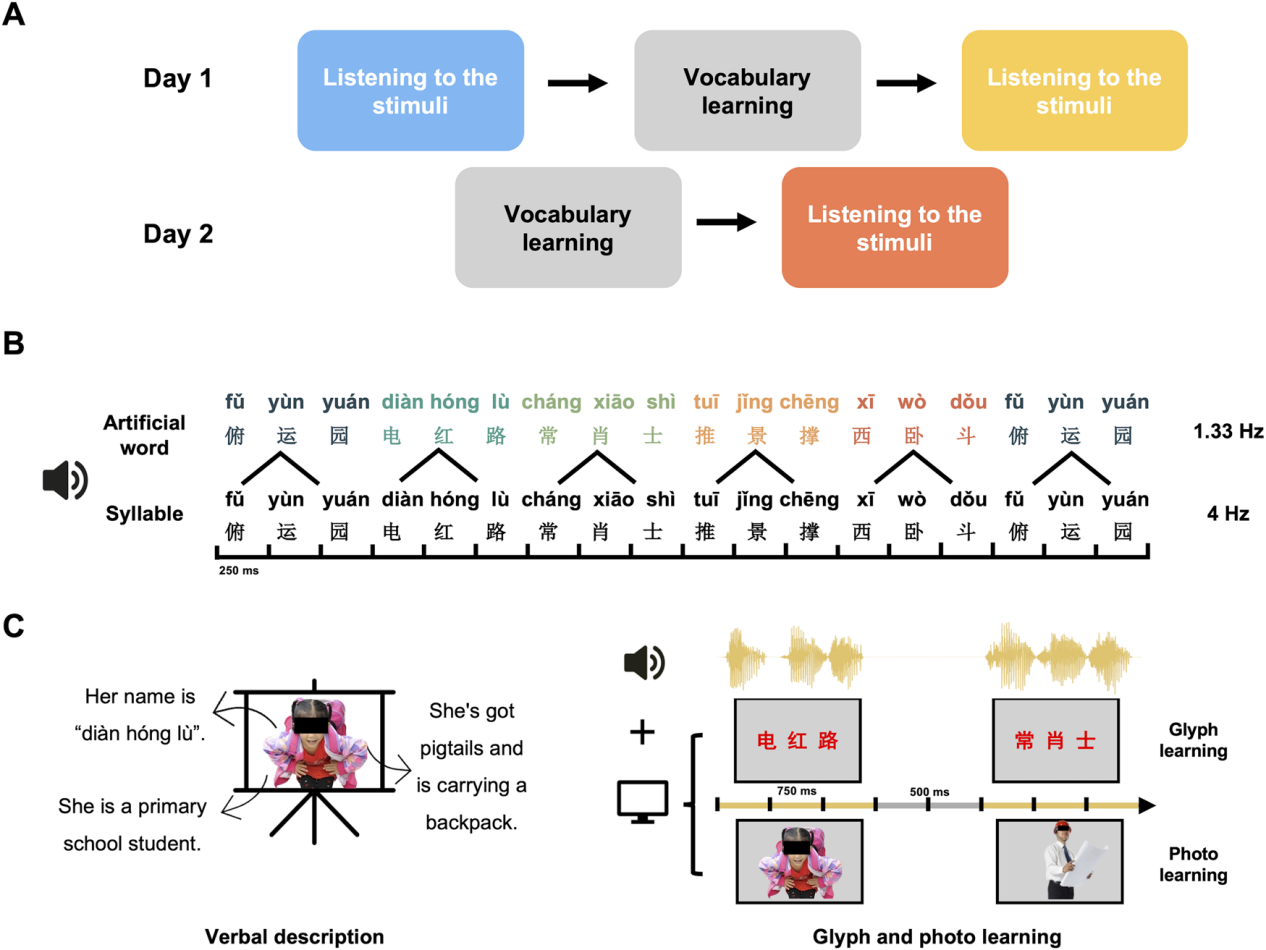
Experimental design. ***A***, Experimental procedures. The experiment was conducted on 2 consecutive days. On the first day, participants underwent three sessions: first, they attentively listened to the stimuli (artificial word sequence). Next, they learned the vocabulary through the verbal description from the experimenter and the audio-visual materials. Finally, as the post-test, they listened to the artificial word sequence again. The second day repeated the first day's vocabulary learning, followed by another session of listening to the artificial word sequence. ***B***, Illustration of speech stimuli presented in the experiment. The stimuli consisted of syllables presented at a constant rate of 4 Hz, with every three syllables forming an artificial word. The presentation rate of the artificial words was 1.33 Hz. The constructed speech sequence consisted of a total of 2,400 syllables, corresponding to 800 artificial words, with a total duration of 10 min. ***C***, During vocabulary learning, five artificial words were learned as five characters (e.g., a girl, an engineer). The entire phase lasted 8 min, including a 2 min verbal description by the experimenter (describing the names, physical characteristics, and occupations of each character), 3 min of glyph learning, and 3 min study of corresponding photos. The learning phase signaled the syllable combinations of each word, which will facilitate the segmentation of continuous speech streams into discrete words after the learning phase. Extended Data Figs. 1-1 and 1-2 display the modulation spectra of the audio envelope and the spectral analysis of simulated neural response waveforms.

The first day of the experiment (learn day 1) consisted of three parts: the artificial words pre-test, vocabulary learning, and the first artificial words post-test (Fig. 1*A*). All participants were naive as to the constituent structure of the stimulus material. In the pre-test phase, baseline EEG data were recorded while participants were instructed to remain still while listening to the stimuli (Fig. 1*B*). To ensure the participants’ attention, a target detection task was conducted. Healthy participants were instructed to press a key if they noticed any syllables that sounded higher in pitch than others (identified as anomalous syllable), while patients were asked to mentally imagine squeezing their right hand. After the pre-test, participants were informed that the audio sequence contained five artificial words that they would learn in the following phase. They were encouraged to concentrate during the learning session and informed that there would be a test afterward to motivate them to do their best. The vocabulary learning session introduced five artificial words as representations of five characters (Fig. 1*C*). This learning phase lasted ∼8 min, including a 2 min verbal description by the experimenter (describing the names, physical characteristics, and occupations of each character), 3 min of glyph learning, and 3 min of photo learning. During the glyph learning and photo learning sessions, each artificial word was played through the headphones, and the corresponding glyphs or photographs were simultaneously displayed on the computer screen. Each word and corresponding screen content lasted 750 ms, with a 500 ms silence and blank screen between each successive artificial word. The five artificial words were repeated 144 times in this manner. It is important to note that the learning phase was designed to facilitate explicit learning of which syllable combinations made up a single word.

10.1523/JNEUROSCI.1684-24.2025.f1-1 Figure 1-1 Download Figure 1-1, DOCX file.

**Figure 2.**
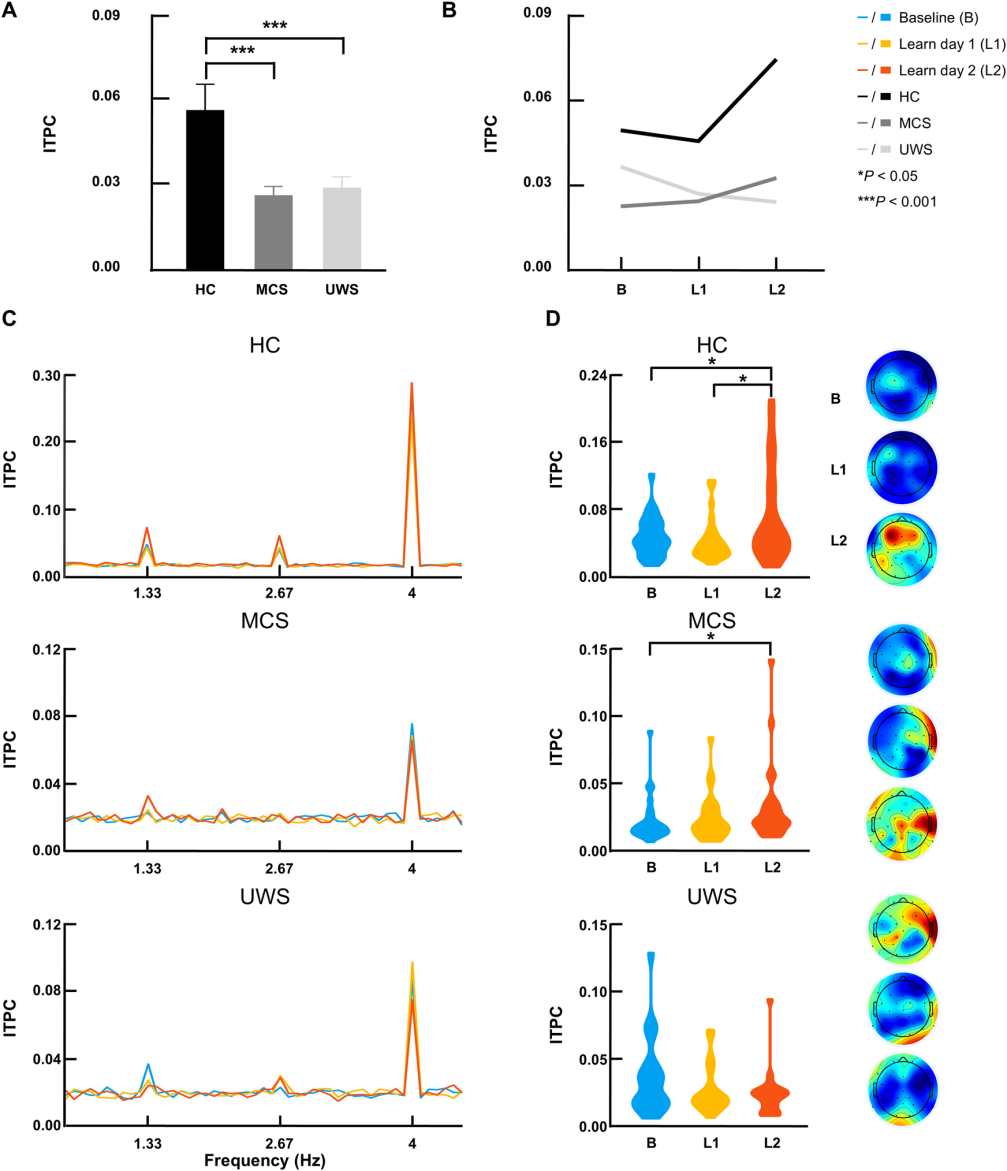
Word-rate ITPC at different learning stages in three participant groups. ***A***, Significant main effect of group on word-rate ITPC with post hoc comparisons. Mean and SEM of word-rate ITPC are depicted. ***B***, The word-rate ITPC as a function of learning stage and group. The significant interaction between learning stage and group indicates that the word-rate ITPC developed differently between the three groups at the different learning stages. ***C***, ITPC spectra of the EEG responses to the different learning stages for the HC and the MCS and UWS groups. Paired *t* test results for ITPC spectral peaks at different learning stages are provided in Extended Data Figure 2-1. ***D***, Word-rate ITPC and EEG topography maps at each learning stage for each participant group. B, baseline; HC, healthy control; L1, learn day 1; L2, learn day 2; ITPC, intertrial phase coherence; MCS, minimally conscious state; UWS, unresponsive wakefulness syndrome; *p < 0.05, ***p < 0.001.

10.1523/JNEUROSCI.1684-24.2025.f1-2 Figure 1-2 Download Figure 1-2, DOCX file.

Following the vocabulary learning phase, participants underwent the first post-test in which they listened to the artificial word sequence again and were instructed to focus on identifying the anomalous syllables and recognizing the newly learned words. After the listening task, participants completed a four-question test of word-feature matching. Healthy participants responded verbally with “yes” or “no,” while patients indicated their responses by imagining clenching their right or left hand.

The second day of the experiment (learn day 2) consisted of vocabulary learning and the second artificial words post-test phase (Fig. 1*A*). Both procedures were consistent with those of the first day.

### EEG recording and preprocessing

Patients were in an open-eyed state during the EEG recording. EEG signals were recorded at a sampling rate of 1,000 Hz with a 32-electrode BrainCap, according to the international 10-20 system (Brain Products). The skin impedance was reduced to <5 kΩ. The electrodes placed on the cheek and on the neck (*n* = 2, i.e., EOG and ECG) were first removed and the rejected channels interpolated using spherical interpolation. The data were then bandpass filtered between a 0.5 and 40 Hz fifth-order zero-phase Butterworth bandpass filter and a 48–52 Hz notch filter. Independent component analysis was performed to remove artifacts related to eye movements and muscle activity. After artifacts removal, data were downsampled to 80 Hz and re-referenced from the initial online reference at FCz to the common average of signals from all channels. All EEG preprocessing steps were performed using the EEGLAB toolbox (version 14.1.2b) running in MATLAB R2021b.

The 10 min continuous data were then segmented into 50 12 s epochs. The epoch length of 12 s was selected based on evidence from previous studies (Staresina et al., 2015; Kang et al., 2018; Sorinas et al., 2019; Chen et al., 2020; Lu et al., 2022) and was aligned with the structure of the experimental stimuli, with each corresponding to the duration of 16 trisyllabic words. All the segmented single-trial EEG data were transformed into the frequency domain using the discrete Fourier transform. Intertrial phase coherence (ITPC) was used to analyze the synchronization of neural responses at specific frequencies. It was defined as follows:

$$\mathrm{ITPC}(f)=\frac{1}{n}\left(\sum_{k\;=\;1}^{n}\cos{\left(\theta_{k}\right)}^{2}+\sum_{k\;=\;1}^{n}\sin{\left(\theta_{k}\right)}^{2}\right).$$

ITPC was calculated using the phase information at each frequency (*f*), which involves using the phase angle (*θ*) of the complex-valued Fourier coefficients, where *n* is the total number of trials. ITPC values range from 0 to 1, with a value closer to 1 indicating a higher degree of neural synchronization and a value closer to 0 indicating less neural synchronization between neurons. The final ITPC value was averaged across all electrodes.

### Statistical analysis

The statistical analysis of the EEG data focused on two frequencies of interest: the syllable presentation rate (4 Hz) and the artificial word-rate (1.33 Hz). Statistical analyses were conducted using IBM SPSS Statistics 26. To explore the neural tracking effects of artificial words in each condition across the two frequencies, we first performed paired *t* tests on the spectral peak values of the ITPC to examine whether the neural responses at each frequency were significantly stronger than the average of the four adjacent frequencies (two on each side).

In the analysis of word learning, we conducted a two-way repeated-measures analysis of variance (ANOVA) on word-rate and syllable-rate ITPC, respectively, with learning stage as the within-participants factor and group as the between-participants factor. Significant main effects were followed by post hoc *t* tests for further analysis. Significant interactions were investigated using one-way repeated-measures ANOVA for each group, with learning stage as the within-participants factor, followed by post hoc comparisons if necessary.

To assess the relationship between the patients’ language learning effects as indicated by the EEG and their clinical behavioral performance at the time of EEG recording, we conducted a two-tailed bivariate Pearson’s correlation analysis in the MCS and UWS groups, separately. Each patient's level of language learning was calculated as the difference between the word-rate ITPC after the second learning session and the word-rate ITPC at baseline (Annen et al., 2019). The highest total score on the CRS-R at the time of the EEG was used as the behavioral score. As a control analysis, Pearson’s correlations were also performed between syllable-rate ITPC and total CRS-R score across patients.

If a significant correlation was observed, we further validated this relationship using the CRS-R index, which is calculated from the highest total score on the CRS-R at the time of the EEG, as the behavioral score. The CRS-R index offers the opportunity to improve the interpretation of clinical assessment relative to individual CRS-R items, as it provides an overall score derived from multiple dimensions which may better reflect the degree of impairment (Annen et al., 2019).

We then used the EEG data to predict the patients’ CRS-R prognosis at 6 months. Pearson’s correlations were calculated between ITPC and CRS-R at 6 month follow-up assessment for word and syllable rates. Partial correlation analysis was also performed to examine the relationship between ITPC and behavioral recovery at 6 months, controlling for behavioral scores at the time of EEG recording. If a significant correlation was observed in these analyses, we also validated the relationship using the prognosis CRS-R index, which was calculated from the CRS-R at 6 month follow-up assessment.

Backward multiple linear regression analyses were conducted to assess the prognostic value of ITPC beyond clinical characteristics. The CRS-R score at 6 months was treated as the dependent variable. Predictor variables included standard clinical parameters (i.e., age, CRS-R score at EEG recording, aetiology, months between injury, and EEG recording) and EEG-specific measures (i.e., ITPC difference between learn day 2 and the baseline at word and syllable rates). The backward method was used, with nonsignificant predictors (*p* > 0.1) sequentially removed until significant predictors (*p* < 0.05) were identified for the best-fitting model.

Finally, we investigated whether narrow-band EEG activity synchronized with vocabulary after explicit learning could potentially serve as a diagnostic and predictive tool for DoC patients. Predictive modeling analysis was performed using the “generalized linear model” method from the caret package (6.0.86) in R (3.6.1). The data were centered and scaled before entering the model. Ten-fold cross validation was used to examine model performance. We conducted a permutation test by shuffling the dependent variables 1,000 times to generate the *p* value of our models. For classification models, we compared the accuracy of the model with random models. For regression models, we compared their root mean square errors with random models. Three diagnostic models were built. First, we classified HC and patients with DoC using the ITPC difference of the word and syllable rates between learn day 2 and baseline. Then we classified different DoC variants (i.e., MCS vs UWS) using the same two ITPC measures. Beyond classification, we further predicted patients’ level of consciousness (i.e., CRS-R index) using the two ITPC difference measures. For prognostic function, we predicted patients’ level of recovery at 6 months (i.e., the CRS-R index change) using the two ITPC measures plus the baseline CRS-R index.

## Results

### EEG word tracking effects

Figure 2*C* depicts the ITPC spectra for the HC, MCS, and UWS groups. We observed clear peaks in the HC group after the learning phase at word (*t*_(24)_ = 4.828, *p* = 6.43 × 10^−5^) and syllable rate (*t*_(24)_ = 7.483, *p* = 1.01 × 10^−7^) compared with adjacent frequencies. For the MCS group, we found a similar peak trend (word: *t*_(24)_ = 2.278, *p* = 0.032; syllable: *t*_(24)_ = 5.421, *p* = 1.44 × 10^−5^). For the UWS group, although the syllable rate peak was observed, the word-rate peak was not obvious (word: *t*_(20)_ = 1.222, *p* = 0.236; syllable: *t*_(20)_ = 3.448, *p* = 0.003). Paired *t* test results for spectral peaks at other learning stages (i.e., baseline and learn day 1) are shown in Extended Data Figure 2-1.

10.1523/JNEUROSCI.1684-24.2025.f2-1 Figure 2-1 Download Figure 2-1, DOCX file.

### EEG word learning effects

We explored EEG evidence of word learning in three groups of participants. The neural tracking responses of words and syllables at different stages of learning were analyzed in the three groups. We investigated whether the learning curve differed in HC, and patients in MCS and with UWS and explored the specific learning effects in each group.

#### Word-rate ITPC

First, the two-way repeated-measures ANOVA (learning stage × group) showed that word-rate ITPC was significantly affected by both learning stage (*F*_(2,136)_ = 4.064, *p* = 0.027) and group (*F*_(2,68)_ = 14.617, *p* = 5.24 × 10^−6^; Fig. 2*A*). A significant interaction effect between learning stage and group was observed for word-rate ITPC (*F*_(4,136)_ = 4.277, *p* = 0.005; Fig. 2*B*). This indicates that the word-rate ITPC developed differently between the three groups at the different learning stages. Post hoc comparisons showed that word-rate ITPC was greater in HC compared with MCS (*p* = 1.63 × 10^−5^) and UWS (*p* = 1.70 × 10^−4^) patients (Fig. 2*A*).

To examine the specific learning effects of each group, a one-way repeated-measures ANOVA was performed. Figure 2*D* shows the word-rate ITPC and its topographical distribution at different learning stages in each group. In the HC group, 60% of participants showed increased ITPC, and the group demonstrated a linear increase in word-rate ITPC with learning (*F*_(2,48)_ = 5.553, *p* = 0.018; linear contrast: *F*_(1,24)_ = 5.606, *p* = 0.026). A significant increase in word-rate ITPC was found after the second learning session compared with baseline (*p* = 0.026) and after the first learning session (*p* = 0.018). Interestingly, 76% of the MCS patients exhibited increased ITPC, and the MCS group also showed an increase in word-rate ITPC as a function of learning stage (*F*_(2,48)_ = 3.037, *p* = 0.072; linear contrast: *F*_(1,24)_ = 5.415, *p* = 0.029), with a significant increase in the last learning stage compared with baseline (*p* = 0.029). In contrast, only 38% of UWS patients showed an increase ITPC, no significant change was observed in this group over the different learning stages (*F*_(2,40)_ = 2.458, *p* = 0.118; linear contrast: *F* = 3.783, *p* = 0.066).

#### Syllable-rate ITPC

**Figure 3.**
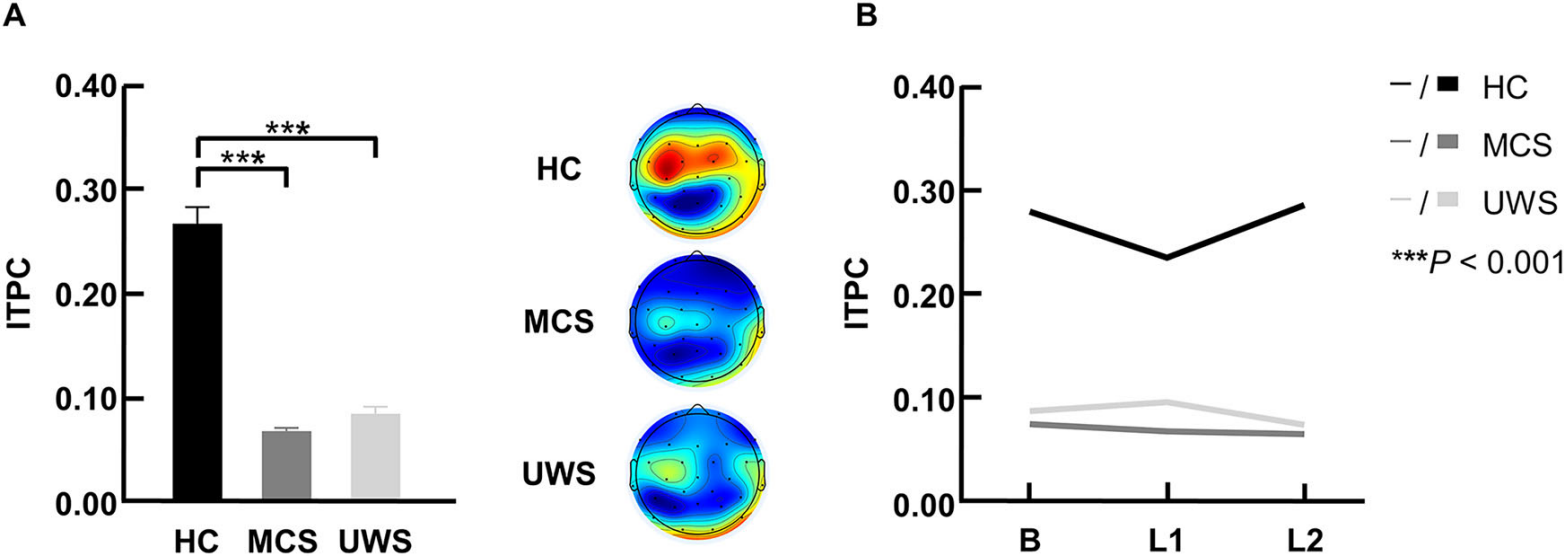
Syllable-rate ITPC at different learning stages in three participant groups. ***A***, Significant main effect of group on syllable-rate ITPC with post hoc comparisons. Mean and SEM of syllable-rate ITPC and EEG topography maps are depicted. ***B***, The syllable-rate ITPC as a function of learning stage and group. No significant interaction between learning stage and group was observed. B, baseline; HC, healthy control; L1, learn day 1; L2, learn day 2; ITPC, intertrial phase coherence; MCS, minimally conscious state; UWS, unresponsive wakefulness syndrome; ***p < 0.001.

The two-way repeated-measures ANOVA (learning stage × group) revealed a significant main effect of group on syllable-rate ITPC (*F*_(2,68)_ = 37.795, *p* = 9.19 × 10^−12^; Fig. 3*A*). No significant main effect of learning stage (*F*_(2,136)_ = 0.717, *p* = 0.476) or interaction (*F*_(4,136)_ = 1.742, *p* = 0.152) was observed (Fig. 3*B*). Post hoc analysis showed a greater syllable-rate ITPC in the HC group compared with the MCS (*p* = 9.11 × 10^−11^) and UWS (*p* = 5.64 × 10^−9^) groups (Fig. 3*A*). Figure 3*A* also shows the topographical distribution of syllable-rate ITPC in each group.

### Relationship between ITPC and behavioral score at the time of the EEG

**Figure 4.**
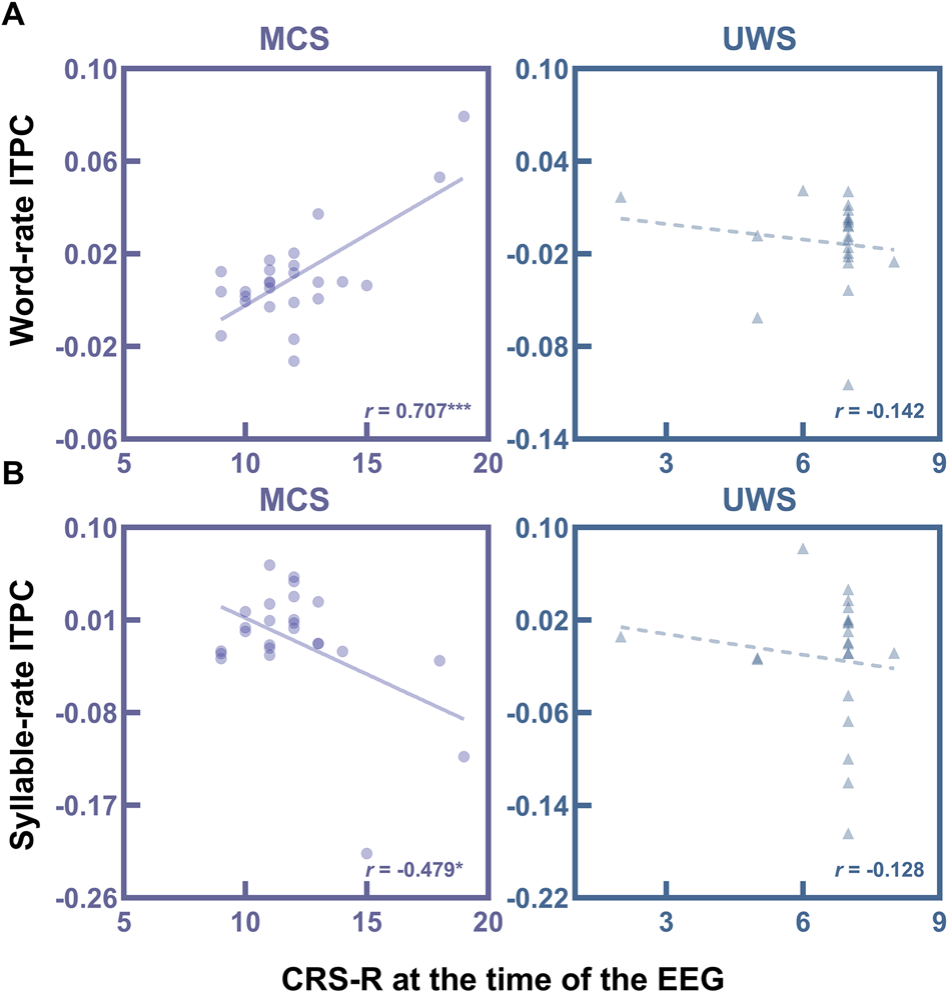
Correlations between ITPC and CRS-R score at the time of the EEG. ***A***, Pearson’s correlation between word-rate ITPC change and CRS-R score at the time of the EEG in MCS and UWS groups. Word-rate ITPC change was calculated as the difference between the word-rate ITPC after the second learning session and the word-rate ITPC at baseline. ***B***, Pearson’s correlation between syllable-rate ITPC change and CRS-R score in MCS and UWS groups. Syllable-rate ITPC change was calculated as the difference between the syllable-rate ITPC after the second learning session and the syllable-rate ITPC at baseline. Extended Data Figure 4-1 provides the correlation between ITPC at different learning stages and CRS-R scores in MCS and UWS patients. Extended Data Figure 4-2 presents ITPC values and behavioral scores for the MCS group. CRS-R, coma recovery scale-revised; HC, healthy control; ITPC, intertrial phase coherence; MCS, minimally conscious state; UWS, unresponsive wakefulness syndrome; *p < 0.05, ***p < 0.001.

As shown in Figure 4*A*, word-rate ITPC change after the learning phase was positively correlated with CRS-R in the MCS group (*r*_(23)_ = 0.707, *p* = 7.84 × 10^−5^). However, no significant correlation was found in the UWS group (*r*_(19)_ = −0.142, *p* = 0.539). For the correlation between the change in syllable-rate ITPC and CRS-R score, a significant negative relationship was found in the MCS group (*r*_(23)_ = −0.479, *p* = 0.015; Fig. 4*B*), which may be related to the antagonism between word-rate and syllable-rate responses. In the UWS group, no significant correlation was observed between syllable-rate ITPC change and CRS-R score (*r*_(19)_ = −0.128, *p* = 0.580). Pearson’s correlation coefficients between raw ITPC at different learning stages and patients’ CRS-R scores are presented in Extended Data Figure 4-1, with the ITPC values and behavioral scores for the MCS group listed in Extended Data Figure 4-2. To ensure the stability of the significant correlations observed, we calculated the correlation between ITPC change and the CRS-R index, and the results remained consistent. Specifically, word-rate ITPC change after the learning phase showed a positive correlation with CRS-R index (*r*_(23)_ = 0.640, *p* = 5.66 × 10^−4^), whereas syllable-rate ITPC change showed a negative correlation with CRS-R index (*r*_(23)_ = 0.423, *p* = 0.035).

10.1523/JNEUROSCI.1684-24.2025.f4-1 Figure 4-1 Download Figure 4-1, DOCX file.

**Figure 5.**
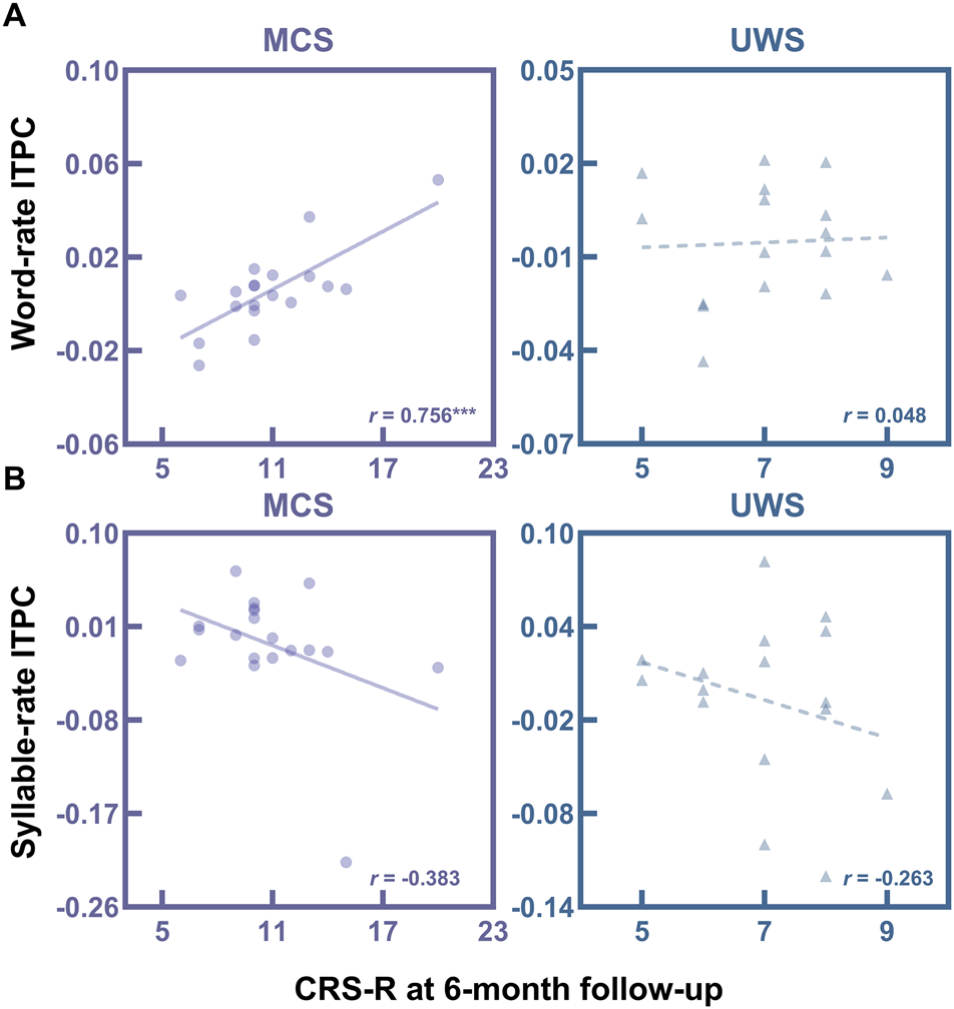
Correlations between ITPC and CRS-R score at 6 months. ***A***, Pearson’s correlation between word-rate ITPC change and CRS-R score at 6 months in MCS and UWS groups. Word-rate ITPC change was calculated as the difference between the word-rate ITPC after the second learning session and the word-rate ITPC at baseline. ***B***, Pearson’s correlation between syllable-rate ITPC change and CRS-R score at 6 months in MCS and UWS groups. Syllable-rate ITPC change was calculated as the difference between the syllable-rate ITPC after the second learning session and the syllable-rate ITPC at baseline. CRS-R, coma recovery scale-revised; HC, healthy control; ITPC, intertrial phase coherence; MCS, minimally conscious state; UWS, unresponsive wakefulness syndrome; ***p < 0.001.

10.1523/JNEUROSCI.1684-24.2025.f4-2 Figure 4-2 Download Figure 5-1, DOCX file.

### Relationship between ITPC and behavioral score at 6 months

#### Correlation between ITPC and behavioral score at 6 months

In the MCS group, the word-rate ITPC change after learn day 2 compared with baseline was significantly correlated with CRS-R scores at the 6 month follow-up assessment (*r*_(23)_ = 0.756, *p* = 1.83 × 10^−4^); no significant correlation was found in the UWS group (*r*_(19)_ = 0.048, *p* = 0.859; Fig. 5*A*). The association in the MCS group remained significant after controlling for the CRS-R score at the time of EEG recording (partial *r*_(22)_ = 0.623, *p* = 0.006). For significant correlations, we further validated the results using the prognosis CRS-R index, confirming a significant positive relationship between the word-rate ITPC change and the prognosis CRS-R index (*r*_(23)_ = 0.737, *p* = 3.22 × 10^−4^), which remained significant after controlling for the CRS-R index (partial *r*_(23)_ = 0.599, *p* = 0.009).

The change in syllable-rate ITPC was not significantly associated with CRS-R scores at the 6 month follow-up assessment in either group (MCS: *r*_(23)_ = −0.383, *p* = 0.106; UWS: *r*_(19)_ = −0.263, *p* = 0.324; Fig. 5*B*).

#### Linear regression modeling predicting behavioral score at 6 months

In all the patients with DoC, the variance in patient prognosis measured by the CRS-R score at 6 months was best explained by a model (*F*_(3,31)_ = 34.416, *p* = 5.47 × 10^−10^; Table 2) that included age (*p* = 0.007), CRS-R score at the time of the EEG recording (*p* = 1.24 × 10^−8^), and the change of the word tracking response after learn day 2 compared with baseline (*p* = 6.05 × 10^−4^). This combination of predictors explained 15.3% more variance in the outcome than a model including only the CRS-R score at the time of EEG recording and 10.8% more variance in the outcome than a model including the CRS-R score and age. The established linear regression model indicates that including the factor of brain activity related to word tracking may increase the explanatory power of the model.

**Table 2.** Linear regression modeling predicting 6 month follow-up CRS-R outcome

Model	Coefficients	Beta (unstandardized)	Beta (standardized)	*t*	*P*	Adjusted *R*^2^	*F*	Significance	df
1	Age	−0.054	−0.240	4.844	0.044	0.758	18.703	1.27 × 10^−8^	6
	CRS-R at EEG	0.701	0.729	−2.114	6.28 × 10^−8^				
	Time since injury	−0.028	−0.057	7.282	0.610				
	Etiology	−0.756	−0.113	−0.516	0.215				
	Syllable-rate ITPC	−7.147	−0.124	−1.269	0.173				
	Word-rate ITPC	52.377	0.312	−1.398	0.004				
2	Age	−0.061	−0.274	−2.986	0.006	0.764	22.971	2.82 × 10^−9^	5
	CRS-R at EEG	0.709	0.737	7.555	2.50 × 10^−8^				
	Etiology	−0.744	−0.111	−1.267	0.215				
	Syllable-rate ITPC	−6.649	−0.115	−1.342	0.190				
	Word-rate ITPC	55.725	0.332	3.640	0.001				
3	Age	−0.060	−0.267	−2.882	0.007	0.759	27.753	1.06 × 10^−9^	4
	CRS-R at EEG	0.711	0.739	7.505	2.29 × 10^−8^				
	Syllable-rate ITPC	−7.861	−0.137	−1.601	0.120				
	Word-rate ITPC	59.571	0.355	3.931	4.62 × 10^−4^				
4	Age	−0.061	−0.274	−2.888	0.007	0.747	34.416	5.47 × 10^−10^	3
	CRS-R at EEG	0.735	0.764	7.655	1.24 × 10^−8^				
	Word-rate ITPC	59.285	0.354	3.817	6.05 × 10^−4^				

Backward linear regression results. Model 1 included all predictors, narrowed down through backward linear regression to Model 4, which achieved the optimal explanation of the variance in the prognosis outcome. Word-rate and syllable-rate ITPC was calculated as the difference between the ITPC after the second learning session and the ITPC at baseline. CRS-R, coma recovery scale-revised; EEG, electroencephalogram; ITPC, intertrial phase coherence.

### The clinical application of the ITPC

To assess the clinical potential of ITPC values, we trained a series of predictive models for the diagnosis and prognosis of DoC (Table 3). Although only two ITPC difference values were included, all three diagnostic models showed significantly good performance: they were able to discriminate patients from HC (ACC = 73%, *p* = 5.00 × 10^−4^), MCS from UWS (ACC = 70%, *p* = 0.004), and predict patients’ specific level of consciousness (*R*^2^ = 0.489, *p* = 0.002). Regarding the effects of the predictors, the difference in word-rate ITPC between learn day 2 and baseline was always significant (beta > 0.691, *p* < 0.02), but not syllable-rate (|beta| < 0.102, *p* > 0.71). Individuals with greater increases in word-rate ITPC after 2 d were more likely to be HC compared with DoC, to be MCS compared with UWS, and to have a higher CRS-R index. For prognosis, the model with ITPC plus baseline level of consciousness significantly predicted 6 month change in consciousness (*R*^2^ = 0.582, *p* = 0.013). Both the word-rate ITPC difference (beta = 6.993, *p* = 0.005) and baseline CRS-R index (beta = −8.945, *p* = 6.07 × 10^−4^) showed significant effects, and syllable-rate ITPC (beta = −4.458, *p* = 0.042) also contributed to the model. Although syllable-rate ITPC was significant, its impact was less pronounced compared with the word-rate ITPC and baseline CRS-R index. Patients with a greater increase in word-rate ITPC and a lower CRS-R index at baseline tended to have a better recovery at 6 months.

**Table 3.** The predictive modeling of ITPC difference for diagnosis and prognosis

	Models			
	HC - DoC	MCS - UWS	Baseline CRS-R index	CRS-R index change
Model performance				
ACC	73%***	70%**	-	-
*R*^2^	-	-	0.49**	0.58*
Predictor effect (beta)				
Word-rate ITPC difference	0.69*	1.40*	12.62***	6.99**
Syllable-rate ITPC difference	0.10	0.01	−4.75	−4.46*
CRS-R baseline				−8.95***

Numbers in the parentheses are the beta values. Word-rate and syllable-rate ITPC difference were calculated as the difference between the ITPC after the second learning session and the ITPC at baseline. ACC, accuracy; CRS-R, coma recovery scale-revised; DoC, disorders of consciousness; ITPC, intertrial phase coherence; MCS, minimally conscious state; UWS, unresponsive wakefulness syndrome.

* *p* < 0.05.

** *p* < 0.01.

*** *p* < 0.001.

## Discussion

In this study, we used a bedside EEG paradigm to record brain activity in response to auditory stimuli (i.e., artificial word speech sequences) in patients with DoC and then performed an analysis of neural tracking activity following an explicit learning paradigm to assess whether these patients retain residual learning abilities by comparing their neural activity patterns with those of HC.

From the characteristics of the neural phase-locking patterns after the learning phase, we observed clear peaks in word-rate in the HC and MCS groups and in syllable-rate in all the participants (i.e., HC, MCS, and UWS groups). Importantly, only the HC and MCS groups exhibited a significant linear increase in word-rate ITPC over the course of the learning phase. Furthermore, we observed a significant positive correlation between EEG changes in the neural response to word-rate after the learning phase and clinical behavioral performance at the time of EEG recording and at 6 months in the MCS group. These results suggest the retention of residual language learning ability in the MCS group. We have also shown the clinical value of EEG signals reflecting language processing ability as a cognitive indicator to predict residual consciousness levels and prognosis in patients with DoC. This new biomarker has the potential for clinical application to improve the diagnosis, prognosis, and treatment of these patients.

### Vocabulary learning and DoC

In recent years, a number of studies have investigated the ability of patients with DoC to track language structure over time as a reflection of their state of consciousness by recording their brain activity (Naci et al., 2014; Wang et al., 2015; Braiman et al., 2018). Sokoliuk et al. (2021) and Gui et al. (2020) conducted EEG studies using multilevel language processing paradigms in acute and chronic patients with DoC, respectively. They found that hierarchical linguistic narrow-band EEG activity could be used to diagnose states of consciousness and predict recovery in patients. In addition, a recent EEG study has shown statistical implicit learning in patients with MCS who exhibited neural tracking of multisyllable words in structured speech sequences (Xu et al., 2022).

Based on these findings, our study used an explicit language learning paradigm to provide additional evidence for the language learning abilities of patients with DoC as a way of inferring their level of consciousness. We hypothesized that the use of an explicit learning paradigm would allow us to better capture any potential learning effects, even in patients with severely impaired brain function. Our results indicate that after the explicit learning paradigm, EEG recordings from the MCS patients showed similar neural activity to HC in tracking word structure. More importantly, we observed a linear increase in neural tracking responses over the course of the learning phase in MCS patients, a trend consistent with what is typically observed in HC. Therefore, our study provides evidence that the learning paradigm can induce enhanced cortical neural tracking of artificial word-rate transitional probabilities in MCS patients. This is consistent with previous neuroimaging studies showing the presence of residual cognitive abilities, such as language processing, in some patients with MCS (Nigri et al., 2017; Aubinet et al., 2020, 2022). Of note, although we used an explicit learning paradigm, the EEG measure cannot directly assess the explicit learning abilities of the participants. Statistical learning inevitably occurs alongside explicit learning and was reflected in the EEG signal (Chen et al., 2020).

However, no learning effects were observed in the patients with UWS. This finding is consistent with a previous study that found the disappearance of responses at the phrase and sentence level in patients with UWS (Gui et al., 2020). It is also consistent with previous research on anesthesia and sleep, which has concluded that once consciousness is disrupted, deep linguistic processing is not maintained (Adapa et al., 2013; Makov et al., 2017).

### Diagnostic and prognostic potential of EEG speech responses

This study also found that the neural phase-locking value in word-rate is closely related to the clinical behavioral performance of patients with MCS, suggesting that the level of transitional probabilities tracking in the learning paradigm serves as a reliable reflection of the patients’ level of consciousness. Of note, after excluding the two patients with the highest behavioral scores, the significant correlation between the word-rate neural responses and behavioral scores disappeared (*r*_(21)_ = 0.193, *p* = 0.377), indicating that these outliers may have influenced the original results. Therefore, this finding should be interpreted with caution. In addition, we observed a significant negative correlation between syllable-rate response and clinical performance in the MCS group. The direction of the results is consistent with previous learning studies, which found that responses to artificial speech increased at frequencies related to pseudowords and their harmonics, whereas an inverse pattern was found for syllable rate (Buiatti et al., 2009; Batterink and Paller, 2017, 2019).

Previous research has indicated that the pattern of EEG activity to external stimuli is a significant predictive indicator of the prognosis of patients with DoC (Bareham et al., 2020; Amiri et al., 2024; Zhuang et al., 2024). Here, we found that learning-related changes in EEG activity correlated with clinical behavioral outcomes after 6 months in patients with MCS. Specifically, minimally conscious patients who showed a greater increase in neural phase-locking in their EEG during vocabulary learning were more likely to show behavioral improvement at the 6 month follow-up assessment. To assess the prognostic value of ITPC indicators in predicting patient outcome beyond traditional clinical variables, we performed a backward linear regression analysis. The results revealed that, compared with traditional clinical prognostic measures, vocabulary ITPC significantly improved the accuracy of prognosis prediction.

In addition, we used ITPC values to train a number of predictive models, exploring the clinical value of EEG indicators in diagnosis and prognosis. Our research showed that the differences in vocabulary ITPC before and after the learning sessions not only effectively discriminated between HC and patients with DoC, between MCS and UWS patient groups, but also had the ability to predict individual clinical behavioral performance. Furthermore, combining EEG indicators with clinical behavioral performance was able to predict changes in patients’ consciousness 6 months after EEG recording. Note that the word-rate ITPC difference between learn day 2 and baseline was always significant among the predictors, but the syllable-rate difference was not. This may be because the word-rate ITPC difference is related to learning and reflects individual language learning ability and level of consciousness. The syllable-rate ITPC, on the other hand, was related to low-level perception and did not require conscious involvement (Hagoort, 2019).

**Figure 6.**
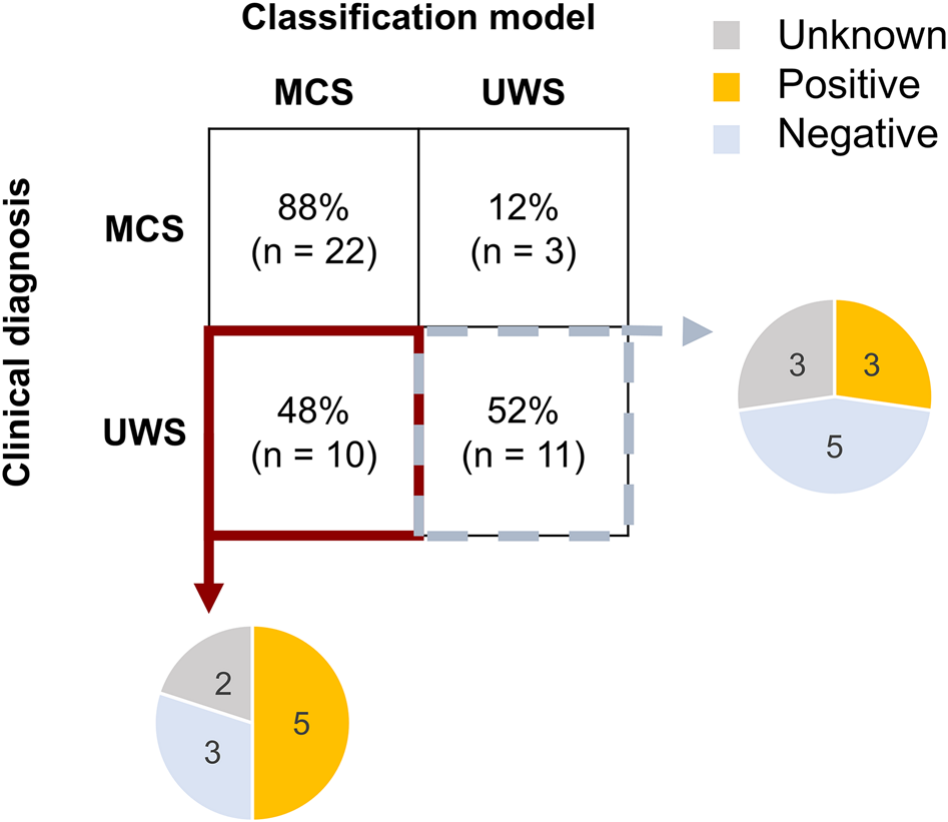
The confusion matrix of diagnosed consciousness classification and 6 month outcomes of patients with UWS. The confusion matrix shows the results of the classification model compared with the CRS-R clinical diagnosis. Of the patients diagnosed with MCS by CRS-R, 88% (*n* = 22) were classified as MCS, while 12% (*n* = 3) were classified as UWS. Among the patients diagnosed with UWS by CRS-R, 48% (*n* = 10) were classified as MCS, while 52% (*n* = 11) were correctly classified as UWS. The pie chart shows the 6 month outcomes of patients diagnosed with UWS.

Interestingly, a subset (48%, 10/21) of UWS patients were classified as MCS in our classification model (Fig. 6). These individuals may have motor impairments that affect their behavioral assessments, potentially leading to misdiagnosis by the CRS-R. Noteworthy, a greater proportion (50%, 5/10) of these potentially misdiagnosed UWS patients showed behavioral improvement 6 months after EEG recording, compared with those consistently diagnosed as UWS by both CRS-R and EEG classifiers (27.3%, 3/11). Future studies could further explore this issue by incorporating other diagnostic tools, such as PET scans and follow-up tests (Stender et al., 2014; Giacino et al., 2018; Aubinet et al., 2020; Kondziella et al., 2020), to assess patients who may have some degree of consciousness that was not detected by the behavioral assessment.

Compared with the diagnostic models, the prognostic model is more complex, taking into account not only word-rate ITPC differences related to language learning, but also CRS-R scores at the time of EEG recording. The baseline level of consciousness may be negatively related to the progress space of consciousness. Therefore, the combination of ITPC and CRS-R may more comprehensively reflect the patient's level of consciousness recovery. In sum, the learning-related difference in word-rate ITPC after the learning phase may be a potential neural marker associated with individual learning processes and provides critical evidence for the future development of diagnostic and prognostic indicators for clinical application. In clinical practice, patients who show high neural phase-locking synchrony in response to vocabulary learning should receive targeted therapeutic interventions, including pharmacological treatments and electrophysiological interventions. The implementation of such targeted interventions may lead to a more efficient use of medical resources for the benefit of patients with DoC.

### Limitations

This study has several limitations. First, we emphasize the small sample size. Future studies should include larger and more diverse samples to confirm the potential of neural tracking during vocabulary learning for diagnostic and prognostic purposes in patients with DoC. In addition, because the same rater did not necessarily perform all five assessments for a given patient, we were unable to calculate inter-rater reliability, which should be addressed in future studies. Another limitation is that our study did not fully isolate explicit learning from implicit learning processes, as the stimuli contained acoustic and phonological confounds that may have influenced neural responses. Future research could use more specific learning paradigms to better discriminate between these processes. Finally, the use of other assessment protocols, such as PET assessment, could validate the diagnostic discrepancy between behavioral and EEG assessments (Kondziella et al., 2016; Marino et al., 2016).

### Conclusion

In conclusion, using a simple auditory bedside EEG paradigm, we observed evidence of improved neural tracking of artificial vocabulary in patients with MCS following an explicit learning paradigm. Simultaneously, EEG indicators based on the level of language learning not only effectively classified patients with DoC but also demonstrated significant accuracy in predicting their prognosis 6 months later. This paradigm has the potential to contribute to both diagnosis and prognosis in this challenging population, thus significantly reducing the prognostic uncertainty in medical decision-making and benefiting the rehabilitation of patients with DoC.
