# Supplementary material for: Clinical Diagnostic and Prognostic Value of Residual Language Learning Ability in Patients with Disorders of Consciousness
Source: J Neurosci. 2025 Apr 17;45(22):e1684242025. doi: 10.1523/JNEUROSCI.1684-24.2025 (PMC12121710; doi:10.1523/JNEUROSCI.1684-24.2025)
Supplement: Figure 1-1 — Download Figure 1-1, DOCX file. [file jneuro-45-e1684242025-s001.docx]

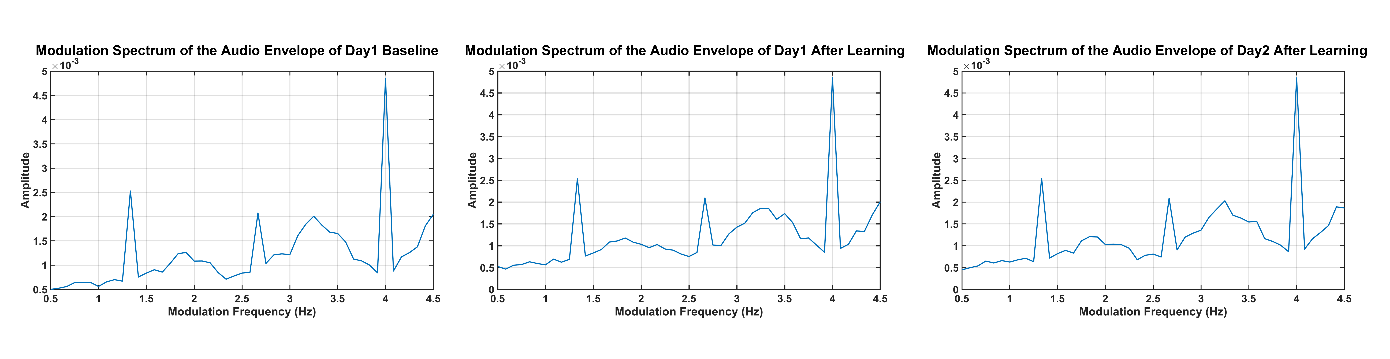
**Extended Data Figure 1-1** **Modulation Spectra of the Audio Envelope Across Different Learning Phases.** The amplitude envelope of the experimental audio stimuli was calculated to examine its modulation spectra. A visible peak at 1.33 Hz was observed, indicating that both the pre- and post-learning experimental stimuli inherently contain acoustic cues.
