# Supplementary material for: Clinical Diagnostic and Prognostic Value of Residual Language Learning Ability in Patients with Disorders of Consciousness
Source: J Neurosci. 2025 Apr 17;45(22):e1684242025. doi: 10.1523/JNEUROSCI.1684-24.2025 (PMC12121710; doi:10.1523/JNEUROSCI.1684-24.2025)
Supplement: Figure 1-2 — Download Figure 1-2, DOCX file. [file jneuro-45-e1684242025-s002.docx]

**
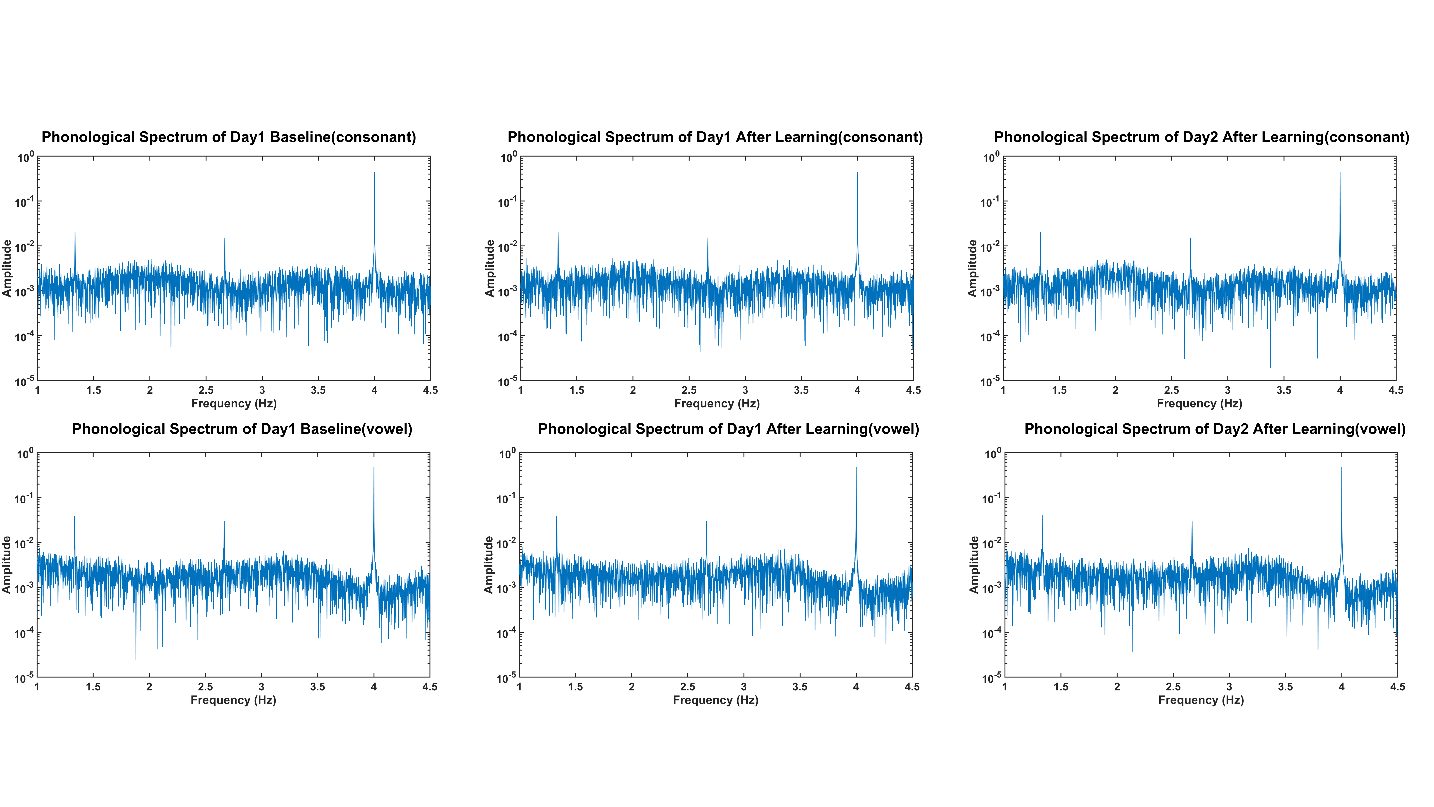
 Extended Data Figure 1-2 Spectral analysis of simulated neural response waveforms based on Phonemic Model.** We assess potential phonological confounds using a Phonemic Model. Specifically, we construct a pulse sequence based on the experimental stimuli. The amplitude of each pulse was assigned according to the consonant or vowel categories, with distinct categories assigned arbitrary values to represent independent neural encoding of phonemic categories. Each word and its constituent syllables were represented by this pulse sequence. The pulse sequence was then convolved with a Gaussian window to simulate neural response waveforms for each syllable. Fast Fourier Transform was used to analyse the frequency distribution of these simulated waveforms. The spectral revealed a clear peak at 4 Hz for the syllable frequency and a peak at 1.33 Hz for the word frequency, indicating phonological regularities in both pre- and post-learning stimuli.
