# Supplementary material for: Clinical Diagnostic and Prognostic Value of Residual Language Learning Ability in Patients with Disorders of Consciousness
Source: J Neurosci. 2025 Apr 17;45(22):e1684242025. doi: 10.1523/JNEUROSCI.1684-24.2025 (PMC12121710; doi:10.1523/JNEUROSCI.1684-24.2025)
Supplement: Figure 2-1 — Download Figure 2-1, DOCX file. [file jneuro-45-e1684242025-s003.docx]

**Extended Data Figure 2-1 Paired *t*-test results for ITPC spectral peaks at different learning stages**

| **Learning stage** | **HC** | |  | **MCS** | |  | **UWS** | |
| --- | --- | --- | --- | --- | --- | --- | --- | --- |
|  | ***t*** | ***P*** |  | ***t*** | ***P*** |  | ***t*** | ***P*** |
| **Word-rate** |  |  |  |  |  |  |  |  |
| Baseline | 6.372 | 1.38×10^-6^*** |  | 0.841 | 0.409 |  | 2.567 | 0.018* |
| Learn day 1 | 5.032 | 3.83×10^-5^*** |  | 1.895 | 0.070 |  | 1.788 | 0.089 |
| Learn day 2 | 4.828 | 6.43×10^-5^*** |  | 2.278 | 0.032* |  | 1.222 | 0.236 |
| **Syllable-rate** |  |  |  |  |  |  |  |  |
| Baseline | 9.117 | 2.89×10^-9^*** |  | 4.029 | 4.89×10^-4^*** |  | 3.342 | 0.003** |
| Learn day 1 | 9.026 | 3.49×10^-9^*** |  | 4.784 | 7.19×10^-5^*** |  | 3.774 | 0.001** |
| Learn day 2 | 7.483 | 1.01×10^-7^*** |  | 5.421 | 1.44×10^-5^*** |  | 3.448 | 0.003** |

For spectral peaks, paired *t*-tests were used to test if the neural response in word or syllable frequency was significantly stronger than the average of the neighbouring four frequency bins. HC = healthy control; MCS = minimally conscious state; UWS = unresponsive wakefulness syndrome; **P* < 0.05; ***P* <0.01; ****P* < 0.001.
