# Supplementary material for: Clinical Diagnostic and Prognostic Value of Residual Language Learning Ability in Patients with Disorders of Consciousness
Source: J Neurosci. 2025 Apr 17;45(22):e1684242025. doi: 10.1523/JNEUROSCI.1684-24.2025 (PMC12121710; doi:10.1523/JNEUROSCI.1684-24.2025)
Supplement: Figure 4-1 — Download Figure 4-1, DOCX file. [file jneuro-45-e1684242025-s004.docx]

**Extended Data Figure 4-1 Correlation between ITPC at different learning stages and CRS-R scores in MCS and UWS patients**

| **Learning stage** | **MCS** | |  | **UWS** | |
| --- | --- | --- | --- | --- | --- |
|  | ***r*** | ***P*** |  | ***r*** | ***P*** |
| **Word-rate** |  |  |  |  |  |
| Baseline | 0.408 | 0.043* |  | 0.181 | 0.433 |
| Learn day 1 | 0.239 | 0.251 |  | 0.119 | 0.606 |
| Learn day 2 | 0.761 | 1.00×10^-5^*** |  | 0.066 | 0.778 |
| **Syllable-rate** |  |  |  |  |  |
| Baseline | 0.501 | 0.011* |  | 0.141 | 0.541 |
| Learn day 1 | 0.410 | 0.042* |  | 0.153 | 0.508 |
| Learn day 2 | 0.185 | 0.376 |  | 0.082 | 0.723 |

MCS = minimally conscious state; UWS = unresponsive wakefulness syndrome; **P* < 0.05; ***P* < 0.01; ****P* < 0.001.
