# Supplementary material for: Clinical Diagnostic and Prognostic Value of Residual Language Learning Ability in Patients with Disorders of Consciousness
Source: J Neurosci. 2025 Apr 17;45(22):e1684242025. doi: 10.1523/JNEUROSCI.1684-24.2025 (PMC12121710; doi:10.1523/JNEUROSCI.1684-24.2025)
Supplement: Figure 4-2 — Download Figure 5-1, DOCX file. [file jneuro-45-e1684242025-s005.docx]

**Extended Data Figure 4-2 ITPC values and behavioural scores for the MCS group**

| **Patient ID** | **Behavioral scores** | **Word-rate ITPC** | **Syllable-rate ITPC** |
| --- | --- | --- | --- |
| MCS01 | 10 | 0.001677021 | 0.002627 |
| MCS02 | 11 | 0.017293635 | -0.01721 |
| MCS03 | 12 | 0.020382447 | 0.047766 |
| MCS04 | 10 | 0.003700378 | -0.00089 |
| MCS05 | 11 | 0.013016788 | -0.02393 |
| MCS06 | 10 | -0.000625468 | 0.018149 |
| MCS07 | 19 | 0.079441523 | -0.12281 |
| MCS08 | 9 | 0.012315763 | -0.02022 |
| MCS09 | 11 | -0.002861619 | 0.025728 |
| MCS10 | 18 | 0.053104979 | -0.02931 |
| MCS11 | 12 | 0.011737653 | 0.051794 |
| MCS12 | 12 | 0.014980862 | 0.033067 |
| MCS13 | 12 | -0.026333028 | 0.010571 |
| MCS14 | 15 | 0.006396538 | -0.217 |
| MCS15 | 11 | 0.007598605 | -0.01426 |
| MCS16 | 14 | 0.007984552 | -0.02033 |
| MCS17 | 11 | 0.007963688 | 0.009643 |
| MCS18 | 13 | 0.007734473 | 0.02774 |
| MCS19 | 9 | 0.003684422 | -0.02251 |
| MCS20 | 12 | -0.001036017 | 0.00192 |
| MCS21 | 13 | 0.037203295 | -0.01255 |
| MCS22 | 13 | 0.00055608 | -0.01284 |
| MCS23 | 11 | 0.00532611 | 0.063443 |
| MCS24 | 12 | -0.016827537 | 0.007172 |
| MCS25 | 9 | -0.015343529 | -0.02732 |
